# Genetic Analysis of the Awn Length Gene in the Rice Chromosome Segment Substitution Line CSSL29

**DOI:** 10.3390/ijms26041436

**Published:** 2025-02-08

**Authors:** Zhengjie Wang, Jun Yang, Tao Huang, Zhihao Chen, Mvuyeni Nyasulu, Qi Zhong, Haohua He, Jianmin Bian

**Affiliations:** 1 College of Agriculture, Jiangxi Agricultural University, Nanchang 330045, China; 18379329405@163.com (Z.W.); junyang2819@163.com (J.Y.); ht19980528@163.com (T.H.); 13970310356@163.com (Z.C.); mvuyeni.nyasulu@gmail.com (M.N.); 18279658618@163.com (Q.Z.); hhhua64@163.com (H.H.); 2 Key Laboratory of Crop Physiology, Ecology and Genetic Breeding, Ministry of Education, Nanchang 330045, China

**Keywords:** *Oryza sativa* L., awn, *An-2*, chromosome segment substitution lines, haplotype

## Abstract

Awn length is a significant agronomic trait in rice. To analyze the genetic mechanism of awn length in the chromosome segment substitution line 29 (CSSL29) derived from 9311 (recipient) into Nipponbare (NIP, donor), an F_2_ segregated population was constructed from 9311 (indica) and CSSL29. The population and candidate genes were analyzed using quantitative trait loci sequencing (QTL-seq), yeast two-hybrid assays, and 3 k and 10 k rice population databases. The results indicated that the awn length in the F_2_ segregating population followed a normal distribution, and the long-awn phenotype in CSSL29 was controlled by multiple genes. Through BSA sequencing data, a major QTL *qAWN4* associated with rice awn length was identified on chromosome 4, containing the cloned gene *An-2*. Further investigation of the CSSL29 long-awn substitution segment revealed the presence of the awn length gene *An-1*, with both genes exhibiting an additive effect on the regulation of the long-awn phenotype. Yeast two-hybrid experiments confirmed no interaction between An-2 and An-1, suggesting that additive effect awn length regulation is not mediated through simple protein-to-protein binding. Population genetic analysis indicated that the *An-2* allele was artificially selected during domestication but did not significantly differ between indica and japonica subspecies. These findings enhance our understanding of the genetic regulation of rice awn length and the domestication of long-awn rice, laying the groundwork for future research in this area.

## 1. Introduction

Rice (*Oryza sativa* L.) is one of the earliest major food crops to be domesticated and serves as a staple food for over half of the global population [1]. During the domestication of rice, traits such as the presence of awns, panicle size, grain shattering, plant height, and seed coat color undergo natural and artificial selection to eliminate negative impacts on human life and agriculture while retaining beneficial traits [2,3,4]. Understanding the development of awns in rice, an important agronomic trait affecting yield, can aid in breeding high-yielding awnless rice varieties [5,6,7]. The awn trait in rice is a quantitative trait controlled by multiple genes [8,9]. Researchers typically use hybrid rice varieties with different awn characteristics to identify and map the genes responsible for awn length. Numerous awn genes and quantitative trait loci have been identified in wild rice and cultivated rice, including indica and japonica, using various genetic approaches such as chromosome segment substitution lines (CSSL), recombinant inbred lines (RIL), and near-isogenic lines (NIL) [10]. By combining QTL analysis with sequencing technology and mutant creation, researchers have identified key genes that regulate awn length.

Combining previous research findings, multiple genes have been identified as participating in the regulation of the growth and development of rice awns. For example, genes such as *An-1*, *An-2*, *LABA1*, *GAD1*, *Awn4-2*, *RAE2*, *RAE3*, and *GAD1-2* have been determined as key genes for rice awn development [11,12,13]. For example, *LABA1* and *An-2* are located on the same QTL on the long arm of chromosome 4, *Awn4-2* and *An-1* are located on the same QTL on chromosome 4, and *GAD1-2* and *GAD1* are located on the same QTL on chromosome 8.

Luo et al. identified the *An-1* gene, which encodes a BHLH transcriptional regulator, and found that it regulates cell proliferation and awn primordia formation, influencing the initiation and elongation of rice awn development [14]. Hua et al. discovered *LABA1*, encoding a cytokinin activating enzyme, through haplotype analysis, suggesting artificial selection during domestication [15]. Gu et al. identified the *An-2*, which enhances cell division and promotes awn elongation [16]. Jin et al. found a frameshift mutation line, *GAD1*, that hinders awn development in cultivated rice [17].

These studies highlight the genetic basis of awn length regulation in rice. Moreover, the awn phenotype can also impact yield traits [7]. For example, previous studies have shown that *OsCKX2* encodes cytokinin oxidase/dehydrogenase, which regulates cytokinin levels in the plant by degrading cytokinins. Suppressing the expression of the *OsCKX2* leads to the accumulation of cytokinins in inflorescence branch mother cells, significantly increasing both the number and length of awns [18,19]. Furthermore, the knockout mutant of *OsCKX2* exhibits several desirable traits: a significant increase in thousand-grain weight, a 40–50% increase in the number of grains per panicle, and improvements in plant height, panicle axis size, and other traits [20]. Significant progress has been made in understanding the genetic control of awn length in rice; however, further research is needed to elucidate the interactions between these genes and their regulatory networks. In-depth studies using diverse rice materials and populations will provide valuable insights into the complex mechanisms underlying awn development in rice.

In this study, a previously constructed broad population of 122 chromosome segment substitution lines was utilized, derived from the cross between the commercial indica variety 9311 as the recurrent parent and a commercial japonica variety NIP as the donor parent. Concurrently, a high-density genetic map was generated through whole-genome resequencing. The map includes 655 bins, spanning a distance of 1480.2 Mb, with an average interval of 0.60 Mb between markers. This population has a higher marker density, effectively meeting the requirements for high QTL mapping resolution. Within this population, a CSSL29 with a long-awn phenotype was identified, and an F_2_ population was constructed using CSSL29 and 9311. Ultimately, a new QTL associated with the long-awn phenotype was identified through QTL-seq technology. Additionally, we demonstrated that *An-1* and *An-2* jointly participate in the regulation of the long-awn phenotype of CSSL29. These findings enhance our understanding of the genetic regulation of rice awn length and the domestication of long-awn rice, laying the groundwork for future research in this area.

## 2. Result

### 2.1. Phenotypic Analysis and Evaluation of Awn

**Figure 1:**
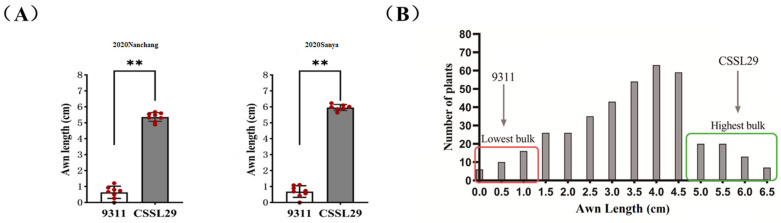
Analysis of seed awn length of parents and F_2_ individuals. (**A**) Comparison of awn length of 9311 and CSSL29 between Sanya and Nanchang in 2020. The double asterisk (**) indicates that *p* is less than 0.01, which means the difference is highly significant, and red dots represent the original data points. (**B**) Histogram of frequency distribution of F_2_ population awn length.

The long-awn type parent CSSL29 has an awn length ranging from 5.5 to 6.5 cm in Sanya City and Nanchang City, while the short-awn type parent 9311 has an awn length shorter than 1 cm in both locations, indicating genetic stability (Figure 1A). To investigate the genetic characteristics of the long-awn trait in CSSL29, awn length statistics were determined in each plant in F_2_ segregated populations of 354. The frequency distribution histogram of awn length in the F_2_ isolate population displayed a normal distribution, indicating that the long-awn phenotype in CSSL29 is a quantitative trait controlled by multiple genes (Figure 1B).

### 2.2. QTL-Seq Data Analysis

**Figure 2:**
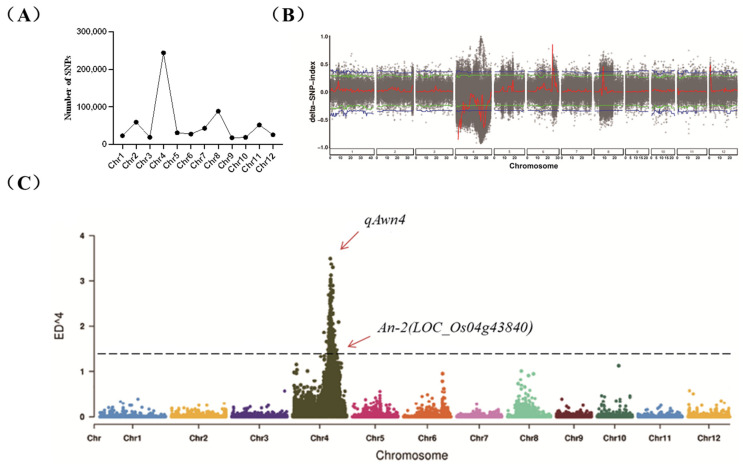
(**A**) Number of SNPs on each chromosome. (**B**) SNP index localization display map. The blue line represents the threshold for the top 1%, the green line represents the threshold for the top 5%, the black dots represent the △SNP-index for each SNP, and the red line represents the result of the sliding window (△SNP-index) analysis. (**C**) Euclidean distance localization display map. The dots represent the SNP ED^4 values, and the black dashed line represents the top 1% threshold.

Sequencing data were filtered to obtain a total of 646,740 high-quality SNPs, primarily enriched on chromosome 4 (Figure 2A). Further analysis of the SNP index and ED results confirmed the presence of a major QTL on chromosome 4, peaking between 23.55 Mb and 26.38 Mb (Figure 2B,C), tentatively named *qAwn4*. The SNP indices between the long-awn mixed pools and the short-awn mixed pools was 0.95 and 0.1, respectively (Table 1). Blast analysis was conducted on the candidate genes within the *qAwn4* interval individually. This analysis identified the gene *LOC_Os04g43840*, which is most likely responsible for the long-awn phenotype observed in the long-awn parent CSSL29. Gene functional annotation analysis revealed the presence of the gene *An-2*, which positively regulates rice grain awn length and encodes a cytokinin-activating enzyme [15]. In addition, analysis using QTL mapping from the previous resequencing of a CSSL population of 9311 NIP revealed that the chromosomal substitution segment containing *An-2* contained another gene related to awn development, *LOC_Os04g28280*(*An-1*), which is a transcription factor [16].

### 2.3. Genetic Analysis of CSSL Population

The results of gene substitution in eight lines within the CSSL population are summarized in Table 2. This analysis focused on two genes, *An-1* and *An-2*, with their respective markers spanning chromosome 4. For *An-1*, the two SNP bin markers M279 and M280, located at positions 16,710,416 bp and 17,414,886 bp, respectively, showed a uniform substitution pattern across lines. CSSL13, CSSL42, CSSL48, and CSSL65 retained the segment from the recurrent parent, 9311 (represented by ‘0’). However, CSSL28, CSSL29, CSSL94, and CSSL122 inherited the segment from the donor parent, NIP (represented by ‘2’). This indicates a consistent introgression of the NIP segment into these five lines for *An-1*. For *An-2*, the markers M297 and M298, located at positions 25,751,684 bp and 26,268,109 bp, displayed a slightly different pattern. The segments for lines CSSL13, CSSL28, CSSL42, CSSL65, CSSL94, and CSSL122 were derived from the 9311 background (‘0’), indicating no substitution. In contrast, CSSL29 and CSSL48 showed introgression from NIP for this gene. CSSL29 exhibited a substitution of NIP segments for both markers, further highlighting its distinct genetic composition for *An-2*.

**Figure 3:**
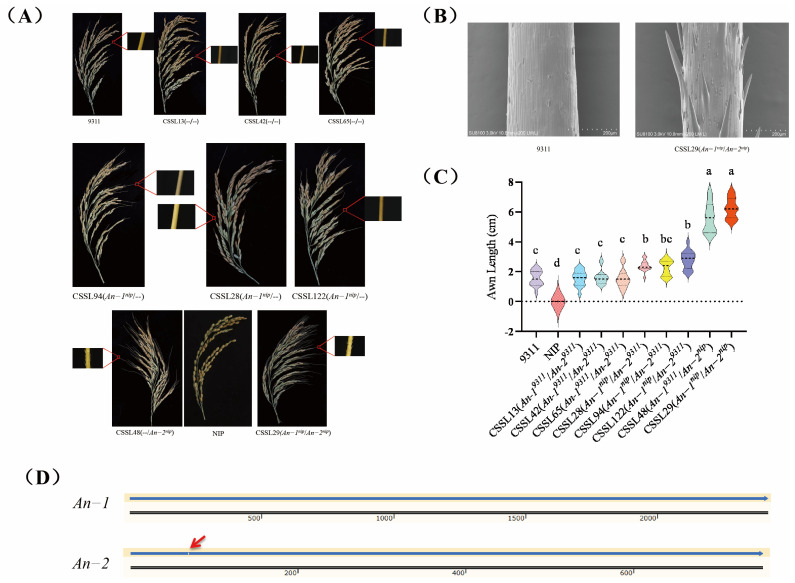
(**A**) Awn length phenotypes of eight lines in CSSL population. (**B**) Comparative SEM images of awn sites in 9311 and CSSL29 seeds. (**C**) Awn length violin map. The middle thick dashed line indicates the median, while the upper and lower thin dashed lines represent the third quartile and the first quartile, respectively, and lowercase letters indicate a significance level of 0.05, and groups marked with different letters have significant differences. (**D**) Sequence comparison of *An-1* and *An-2* in 9311 (top) and NIP (bottom), with red arrows indicating 1 bp deletions.

The results indicate that the substitution of *An-2^nip^* into the 9311 background resulted in significantly elongated grain awns with prickles on the surface, as observed in lines CSSL29 and CSSL48 (Figure 3B). A comparison revealed that the awn length of CSSL48, which solely contained the *An-2^nip^* gene, was slightly shorter than that of CSSL29. The awn lengths of CSSL28, CSSL94, and CSSL122, lines containing only the *An-1^nip^* gene, were shorter than CSSL48 and CSSL29, with smooth awn surfaces. The lines CSSL13, CSSL45, and CSSL65, which did not contain the two genes, exhibited awn lengths similar to the parent 9311 (Figure 3A,C). These results demonstrate that both *An-2^nip^* and *An-1^nip^* influence 9311, and that the combined effect of *An-1* and *An-2* in regulating rice awn length is significantly greater than that of *An-1* alone in regulating the long-awn phenotype.

This finding aligns with previous research indicating that *An-1* and *An-2* have additive effects on rice awn length regulation [16,21]. Comparison of the coding sequences of *An-2^nip^* and *An-2^9311^* revealed a 1 bp deletion in the first exon region of *An-2^9311^*, leading to the premature termination of translation, potentially explaining why 9311 did not exhibit the long-awn and prickle phenotype [15] (Figure 3D). Furthermore, comparison showed that *An-1^nip^* shared the same DNA sequence as *An-1^9311^*, yet *An-1* exhibited different awn lengths in 9311 and NIP, suggesting that *An-1*’s control of rice awn length formation may be influenced by variations in the cis acting element or genetic background. This implies that even with identical gene sequences, different genetic backgrounds can result in phenotypic variations by impacting gene expression or other regulatory mechanisms.

### 2.4. Relationship Between An-1 and An-2

**Figure 4:**
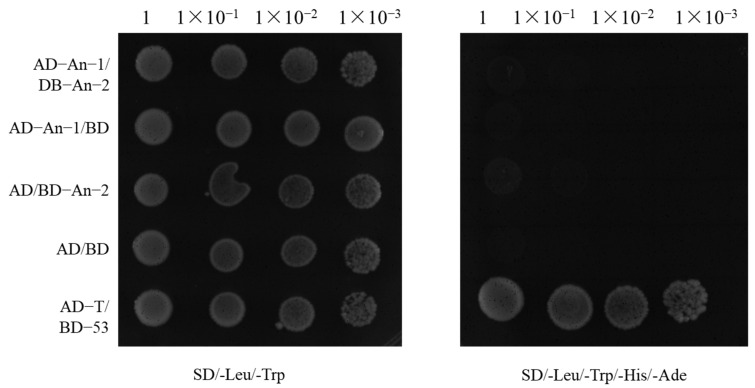
Yeast two-hybrid point-to-point validation plot.

To investigate the potential direct interaction between *An-1* and *An-2*, a yeast two-hybrid experiment was conducted [22,23]. The experimental procedure involved cloning the coding sequences of *An-1* and *An-2* into the yeast two-hybrid vectors pGADT7 and pGBKT7, respectively [24]. This approach allowed for testing the physical interaction between the encoded proteins in yeast cells. The results revealed that only the positive control group (AD-T/BD-53) showed white colonies on SD (-Leu/-Trp/-His/-Ade) selective medium, indicating that BD-An-2 did not exhibit self-activating properties. Co-transformation of AD-An-1 and BD-An-2 did not activate the reporter gene, confirming the absence of a direct interaction between the two proteins (Figure 4). In conclusion, *An-1* and *An-2* operate through distinct mechanisms to regulate rice awn length, suggesting no significant functional interaction between them. This discovery provides the valuable conclusion that these genes do not exert additive effects through simple protein interactions to control rice awn length.

### 2.5. Haplotype and Population Genetic Analysis

**Figure 5:**
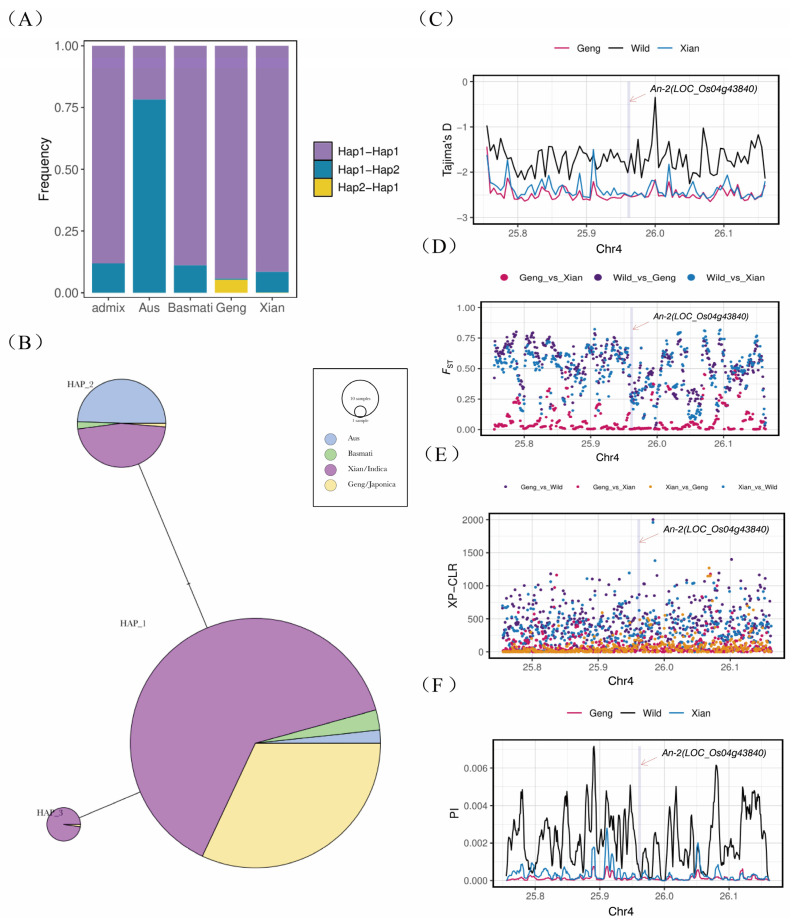
Haplotypes and population genetic analysis. (**A**) Promoter–CDS haplotype frequency distribution plot. (**B**) Haplotype network. (**C**) Fst: index of genetic differentiation between populations. (**D**) Tajima’s D: neutrality test. (**E**) PI: population nucleotide diversity analysis. (**F**) XP-CLR: cross-population composite likelihood ratio test.

The genetic variation in the rice awn length gene *An-2* was analyzed in 3010 resequenced Asian rice populations. The study identified three main haplotypes of the *An-2* gene, with Hap_1 being the predominant haplotype. Notably, the promoter region and coding sequence of *An-2* exhibited two distinct haplotypes, with the Hap_2 haplotype in the promoter region being specific to japonica rice (Figure 5A,B). Population genetic analysis revealed a high fixation index (F_ST_) in the *An-2* region, indicating significant population differentiation during the domestication process from wild rice to cultivated rice (Figure 5C). However, the genetic differentiation between the two cultivated rice subpopulations, indica and japonica, was relatively low. Tajima’s D value was negative, suggesting the presence of numerous low-frequency allelic loci in the *An-2* region. Furthermore, the Tajima’s D value was lower in cultivated rice compared to wild rice, indicating reduced genetic diversity in the *An-2* region in cultivated rice (Figure 5D).

Nucleotide diversity analysis demonstrated a significant reduction in nucleotide diversity in the *An-2* region of cultivated rice compared to wild rice, consistent with the Tajima’s D results, indicating strong selection pressure on the *An-2* region in cultivated rice (Figure 5E). The cross-population composite likelihood ratio test (XP-CLR) confirmed the strong selection pressure on *An-2* in cultivated rice, with a weaker selection signal observed between the indica and japonica subpopulations (Figure 5F). Overall, the study revealed that the *An-2* region underwent artificial selection during rice domestication, with selection and retention of the *An-2* alleles that regulate short or awnless phenotypes.

## 3. Discussion

Elongated rice awns serve to protect seeds from bird predation and aid in seed dispersal and reproduction [9,25]. Studying the genes that regulate rice awn length through cloning and functional analysis can provide insights into the molecular mechanisms behind awn formation and the loss of awn traits during rice domestication. Researchers typically create population materials by hybridizing rice varieties with different awn properties, such as wild and cultivated rice or indica and japonica rice, to locate the QTLs controlling awn genes in rice. By combining sequencing technology and mutant creation, the main effector genes controlling length can be identified [8,26].

With the advancement of sequencing technologies, the QTL-seq method, which leverages pooled sequencing, has been increasingly utilized for the rapid and precise localization of QTLs and significant genes controlling various agronomic traits in rice [27,28,29]. This approach bypasses the laborious process of genotyping each individual from a large mapping population, thereby not only accelerating the identification of candidate genes or QTLs but also significantly reducing the time required for population construction [28,30]. This study delves into the complex genetic framework that controls rice awn length, a trait with both evolutionary and agronomic significance. The identification of the major QTL *qAwn4* on chromosome 4, along with the characterization of *An-2* and *An-1*, reveals a multifaceted genetic control system involving independent yet synergistic mechanisms. These findings highlight the intricate interplay among multiple genes, cis-regulatory elements, and environmental factors in shaping quantitative traits like awn length.

The discovery of An-2 as a cytokinin-activating enzyme influencing awn development aligns with previous research implicating hormonal pathways in rice morphology regulation [16]. Cytokinins play a dual role in growth and differentiation, and the precise modulation by *An-2* may be a key factor in awn elongation [15]. This functional specificity is particularly evident in the CSSL29 background, where the introgression of the *An-2* allele from NIP resulted in elongated, prickle-bearing awns. Additionally, the additive interaction between *An-2* and *An-1*, a transcription factor, provides new evidence of combinatorial genetic effects that can enhance phenotypic expression [14]. Although the yeast two-hybrid assays confirmed no direct protein-protein interaction between An-1 and An-2, functional crosstalk may still occur through shared downstream pathways or transcriptional networks. The additive effects observed in the CSSL population suggest that these genes may converge on overlapping developmental processes or interact indirectly through regulatory hierarchies. Future studies utilizing transcriptomic and proteomic analyses could elucidate these connections, shedding light on how hormonal and transcriptional pathways integrate to govern awn phenotypes. Population genetic analysis of *An-2* across diverse rice accessions offers profound insights into the evolutionary forces shaping this trait. The reduced genetic diversity and high fixation index within the *An-2* locus in cultivated rice suggest strong artificial selection during domestication. These selections likely favored alleles promoting shorter or absent awns, optimizing traits such as seed retention and mechanical harvesting. The differentiation in the promoter regions of *An-2* haplotypes indicates that cis-regulatory evolution, rather than coding sequence variation, drove phenotypic divergence, emphasizing the role of non-coding regions in evolutionary processes. The differential expression and phenotypic outcomes of *An-1* in the 9311 and NIP genetic backgrounds highlight the significant influence of genetic context on gene functionality, but it is also very likely that there are differences in the cis-regulatory elements. Despite the identical coding sequences for *An-1*, the variation in awn length underscores the impact of epistatic interactions and genetic modifiers. These modifiers, potentially located in unlinked loci or regulatory elements, may fine-tune the expression of *An-1*, altering its phenotypic effects [31]. This observation raises questions about the interplay between major and minor genetic determinants in quantitative trait expression. The possible independent regulatory mechanisms of *An-1* and *An-2*, along with their additive effects, demonstrate the polygenic nature of awn length control. The evolutionary divergence in regulatory elements and the differential selection pressures between wild and cultivated rice populations emphasize that domestication involves both natural and artificial selection. The trade-offs between functional utility (e.g., seed dispersal in wild rice) and agronomic practicalities (e.g., harvesting efficiency in cultivated rice) are evident in the retention of specific alleles.

This study not only enhances our understanding of the genetic basis of awn length but also paves the way for investigating the genetic underpinnings of other complex traits in rice. The identification of key genes and their interactions provides a solid foundation for marker-assisted selection and genome editing strategies aimed at improving awn-related traits. Furthermore, the implications of cis-regulatory evolution and genetic context in shaping phenotypic diversity warrant further exploration, particularly in the context of environmental adaptability and crop resilience. In conclusion, the genetic regulation of awn length exemplifies the complexity of polygenic traits, additive effects, and regulatory evolution. These findings underscore the delicate balance between genetic stability and flexibility in trait development, offering valuable insights for evolutionary biology and practical rice breeding.

## 4. Materials and Methods

### 4.1. Test Materials

#### 4.1.1. Plant Materials and Field Treatments

Indica rice variety 9311 and japonica rice variety NIP were obtained from Jiangxi Agricultural University (Nanchang, China) and used as female and male parents, respectively, to generate an F_2_ segregating population of 354 individuals. DNA from the leaves of the parents and from 354 F_2_ individuals at the heading stage was extracted for the subsequent experiments. Lines CSSL13, CSSL28, CSSL29, CSSL42, CSSL48, CSSL65, CSSL94, and CSSL122 were all derived from the 9311–NIP chromosome segment substitution line population constructed earlier by the research group [32] (Table 2). Among them, the CSSL population was obtained by crossing 9311 and NIP to obtain the F_1_ generation, followed by multiple backcrosses and self-crosses using 9311 as the recurrent parent to finally obtain the permanent CSSL population at the BC_3_F_6_ generation. All rice materials were planted in Sanya, Hainan, and Nanchang, Jiangxi, under normal field water and fertilizer management conditions for two seasons. Rice seedlings were transplanted one month after sowing at a density of 10 plants per row, with a row spacing of 40 cm and a plant spacing of 20 cm.

#### 4.1.2. Strains and Vectors

Yeast Y2HGold competent cells: used for yeast two-hybrid experiments, this strain contains reporter genes for the GAL4 activation domain (AD) and GAL4 binding domain (BD) and is capable of growing on selective media, used for detecting protein-protein interaction. *E. coli* competent cells, DH5α: used for plasmid amplification and transformation. DH5α is a commonly used competent cell strain with high transformation efficiency and a stable plasmid maintenance capability. The Y2HGold and DH5α strains were purchased from Tolo Biotechnology (Wuhan, China).

The pGADT7 vector: used for cloning the coding sequence of the target gene to construct a GAL4 activation domain fusion protein. The pGADT7 vector contains the GAL4 activation domain and the T7 promoter, which can drive the expression of the target gene. This vector also contains an ampicillin resistance gene for screening positive colonies. The pGBKT7 vector: used for cloning the coding sequence of the target gene to construct a GAL4 binding domain fusion protein. The pGBKT7 vector contains the GAL4 binding domain and the T7 promoter, which can drive the expression of the target gene. This vector also contains a kanamycin resistance gene for screening positive colonies. The pGADT7 and pGBKT7 vectors were preserved by the Key Laboratory of Crop Physiology, Ecology and Genetic Breeding of the Ministry of Education, Jiangxi Agricultural University.

#### 4.1.3. Experimental Design

Vector construction: the coding sequences of *An-1* and *An-2* were, respectively, cloned into the pGADT7 and pGBKT7 vectors to construct the fusion proteins AD-An-1, AD-An-2, BD-An-1, and BD-An-2. Restriction enzymes and recombinases were used for cloning. The cloned products were transformed into competent *E. coli* cells and cultured in corresponding antibiotic-containing media. Colony PCR and sequencing were then performed to verify the correctness of the cloning, ensuring the accurate insertion and expression of the target genes.

Yeast transformation: the constructed AD-An-1 and BD-An-2 plasmids were co-transformed into the Y2HGold yeast strain using the LiAc/SS-DNA/PEG method, and positive colonies were selected on SD (-Leu/-Trp) double dropout medium to ensure correct plasmid transformation. Subsequently, the positive colonies from the double dropout medium were transferred to SD (-Leu/-Trp/-His/-Ade) quadruple dropout medium for cultivation, and the growth of the yeast strains was observed.

### 4.2. Grain Awn Length Measurement

Once the rice matured, 5 panicles were randomly selected from each F_2_ individual in the field, 10 grain awn lengths were measured for each panicle, and the average value was used as the grain awn length of the F_2_ individual.

### 4.3. Whole-Genome Resequencing

The Illumina HiSeq 4000 platform (The manufacturer of the Illumina HiSeq 4000 is Illumina, Inc., which is headquartered in San Diego, CA, USA) was utilized for the whole-genome resequencing of the high-mixing pool and low-mixing pool created from the F_2_ extreme individuals and the DNA libraries of the parents. The high-mix pool had 97.25 million short reads (150 bp in length), totaling 14.59 Gb, while the low-mix pool had 96.08 million short reads, totaling 14.52 Gb. The parents CSSL29 and 9311 had 95.8 million and 95.72 million short reads, totaling 14.37 Gb and 14.36 Gb, respectively. Using the NIP genome database (http://rice.plantbiology.msu.edu/pub/data/Eukaryotic_Projects/o_sativa/annotation_dbs/pseudomolecules/version_7.0/all.dir/all.con (accessed on 5 January 2020)) as a reference, the end sequences from the extreme-mixed pools and the parents CSSL29 and 9311 were compared, resulting in successful pairing percentages of 91.56%, 92.45%, 91.67%, and 91.81%, respectively (Table 3). This indicated a good sequencing quality, suitable for subsequent QTL-seq analysis.

### 4.4. QTL-Seq Analysis

Thirty individual plants with grain awn lengths shorter than 0.5 cm (short awn) and longer than 4 cm (long-awn) were selected as experimental materials. DNA was extracted from each plant and mixed in equal proportions. Bulk samples representing the highest and lowest awn lengths, as well as the DNA of the two parents, were prepared and sent to Wuhan BGI Technology Co., Ltd. (Wuhan, China), for bulk sequencing using the Illumina HiSeq4000 sequencer [33,34,35]. After filtering out low-quality sequence data, Burrows Wheeler Aligner (BWA) and GATK3.4 software are utilized for reference genome alignment and SNP variation detection [36,37]. The parental genotype frequency at all SNP segregation sites for each mixed offspring pool was calculated using the control parent as a reference, known as the SNP index. The ΔSNP index was then calculated through sliding window analysis, and QTL candidate regions were identified based on the difference in gene frequencies in the population (ΔSNP index) and the ED value (Euclidean distance) to select regions beyond the 99% confidence level [38,39]. The Δ-SNP index was used as an indicator in QTL-seq analysis to identify genomic regions associated with phenotypes. Genes encoded by SNPs in the candidate QTL region were analyzed to discover candidate genes related to the control of rice awn length.

### 4.5. Yeast Two-Hybrid Assay

Yeast two-hybrid assays were conducted with the Y2H Gold yeast strain using the GAL4 two-hybrid system [40] (ToloBio, Wuhan, China), following the manufacturer’s instructions. The pGADT7 and pGBKT7 vectors were used for plasmid constructions. Double dropout plates lacking Trp and Leu were used to select co-transformed colonies. Protein interactions were detected by measuring the growth of colonies on QDO plates lacking Trp, Leu, Ade, and His.

### 4.6. Data Analysis of Rice Population Resources

In the haplotype analysis performed in the 3K Rice Germplasm Project, all single nucleotide polymorphisms (SNPs) of the coding sequence region (CDS) of a specific gene were downloaded from the RFGB v2.0 database, excluding synonymous SNPs. Major haplotypes were defined based on SNP combinations occurring in at least 30 rice varieties. The geneHapR software version 1.2.4 package of R language was used to construct a gene haplotype network in the Rice Super-Population Variation Map database. Population genetic analysis was conducted on 3359 japonica, 5295 indica, and 412 wild rice genome sequences in the Rice Super-Population Variation Map database. VCFtools software (version 0.1.16) was used to calculate the nucleotide diversity (π) and interpopulation fixation index (Fst) within a 200 kb region on each side of the gene, with a 5 kb window and a 1 kb step size. Likewise, Tajima’s D values within each 5 kb window were calculated to assess whether the genetic variation corresponded to neutral evolutionary expectations. In addition, the cross-population composite likelihood ratio test (XP-CLR) scores were calculated for each window by applying the Python 3.0 package XPCLR with a 5 kb window, a 1 kb step size, and a linkage disequilibrium (ld) threshold of 0.95. XP-CLR, as a statistical method for detecting imprints of natural selection in genomes, compares patterns of genetic variation across populations to identify gene regions under selection [41,42].

### 4.7. Statistical Analysis

The phenotype data were analyzed using GraphPad Prism9.5 software and Microsoft Excel 2010. Pairwise comparisons were conducted using the two-sample *t*-test to assess differences between treatment groups. A significance level of 0.05 was used as the threshold for statistical significance. Genomic data analyses and visualization including delta SNP index and Euclidean distance were performed in R software version 4.2.2, using the rMVP package version 1.3.0. The delta SNP index was used to identify the genomic regions associated with awn length, while Euclidean distance was used to assess the genetic divergence between groups.

## Figures and Tables

**Table 1:** Results of QTL detected for grain awn length in rice.

QTL	Chr.	Start	End	Interval (bp)	Δ(SNP Index)	SNP Index	SNP Index
*qAwn4*	4	23,547,272	26,376,809	2,829,537	0.85	0.95	0.10

**Table 2:** Gene substitution in eight lines in the CSSL population.

Gene	Marker	Chr.	Pos	13	28	29	42	48	65	94	122	NIP	9311
*An-1*	M279	4	16,710,416	0	2	2	0	0	0	2	2	2	0
	M280	4	17,414,886	0	2	2	0	0	0	2	2	2	0
*An-2*	M297	4	25,751,684	0	0	2	0	2	0	0	0	2	0
	M298	4	26,268,109	0	0	2	0	2	0	0	0	2	0

Note: ‘0’ means the segment is from 9311, ‘2’ means the segment is from NIP.

**Table 3:** Mapping quality statistics table.

Sample	No. of Clean Reads (Million)	Clean Base (Gb)	Depth	Properly Paired Reads (million)	Properly Paired Ratio
Highest bulk	97.25	14.59	38.957×	89.04	91.56%
Lowest bulk	96.08	14.52	38.782×	89.51	92.45%
CSSL29	95.80	14.37	38.375×	87.82	91.67%
9311	95.72	14.36	38.340×	87.88	91.81%

## Data Availability

The original contributions presented in this study are included in the article. Further inquiries can be directed to the corresponding authors.

## Abbreviations

SNP	Single Nucleotide Polymorphism
QTL	Quantitative Trait Locus
BSA	Bulk Segregant Analysis
GO	Gene Ontology
KEGG	Kyoto Encyclopedia of Genes and Genomes
ED	Euclidean Distance
RIL	Recombinant Inbred Lines
CSSL	Chromosome Segment Substitution Lines
NIL	Near Isogenic Lines
LD	Linkage Disequilibrium
